# Characterization of the Bacterial Community of Rumen in Dairy Cows with Laminitis

**DOI:** 10.3390/genes12121996

**Published:** 2021-12-16

**Authors:** Jian Guo, Ruiying Mu, Shuang Li, Naisheng Zhang, Yunhe Fu, Xiaoyu Hu

**Affiliations:** 1 Department of Clinical Veterinary Medicine, College of Veterinary Medicine, Jilin University, Changchun 130062, China; guojiancloud@sina.com (J.G.); lis19@sina.com (S.L.); zhangns@jlu.edu.cn (N.Z.); fuyunhesky@163.com (Y.F.); 2 Linqu County Animal Husbandry Development Center, Linqu 262600, China; muruiyingwenzhang@163.com

**Keywords:** ruminal microbiota, bovine laminitis, LPS, lactic acid

## Abstract

Laminitis is the inflammation of the lamella, and it has caused great economic loss to the dairy industry and attracted wide attention around the world. In recent years, microbiota are considered to play a significant role in various diseases processes. Therefore, our study aimed to explore the characteristics of ruminal microbiota in laminitis cows. The serum of bovines with or without laminitis was collected to detect concentrations of lipopolysaccharide (LPS), lactic acid, and histamine, and ruminal fluid was collected for 16S rDNA sequence analysis. The results showed a significant increase in LPS and lactic acid levels in the laminitis group compared to the control group cows. In addition, a higher abundance of *Candidatus Saccharimonas*, *Saccharofermentans*, *Erysipelotrichaceae UCG-009* genus, *Acetobacter pasteurianus*, *Clostridium papyrosolvens*, *Ruminococcaceae bacterium AE2021*, *Porphyromonas crevioricanis*, *Pseudomonas boreopolis*, *Pseudomonas psychrotolerans*, *Rothia nasimurium*, and *Rothia pickettii* was detected in the rumen fluid of laminitis bovines. In conclusion, this article confirms that there are differences in rumen microbiota between healthy and laminitis bovines. The elevated abundance of bacteria that enrich acid-enhancing metabolites, as well as increase the concentration of lactic acid and LPS, could be harmful factors to bovines and increase the risk of laminitis.

## 1. Introduction

In the dairy industry, metabolic diseases are a primary health problem, including foot disease [1]. Laminitis, an aseptic inflammation of the sensitive lamina of the hoof, is one of the key causes of major economic losses [2]. In bovines, it can be categorized as acute, subclinical, and chronic laminitis according to the severity and duration of the incident [3]. In the stage of acute and subacute laminitis, the cows are crippled in all their limbs [4,5]. The hoof is elongated into a flattened and broadened shape, named “slipper foot” [6,7]. Changes in postures and locomotion are insignificant and also occur after two to three months of sole ulcers and white-line disease [8,9]. Although laminitis has attracted enough attention, its physiopathology remains unelucidated. Currently, it is commonly considered as the result of the interaction of various factors, including breeding conditions and metabolic insults, which increase the accumulation of toxic substances, such as histamine, lactic acid, and lipopolysaccharide (LPS), leading to vascular lesions and degradation of the suspensory apparatus of the third phalanx within the digit [10,11,12].

Host–rumen–microbe interactions play a critical role in the maintenance of physiological activity and are therefore a contributing factor affecting the health of dairy cows [13]. It is well established that the disturbance of ruminal microbiota can induce bovine laminitis through indigestion caused by ruminal acidosis [14]. Overuse of the concentrates aiming at increasing milk yield contributes to the imbalance of ruminal flora, and increasing harmful bacteria could produce more endotoxins and trigger the release of histamines and lactic acid [15,16,17]. Inflammation reactions are the main pathological changes in the incipient stage of laminitis, and the effects of LPS on laminitis have been examined, such as an intradermal injection of LPS into bovines to induce laminitis [18]. In ruminal acidosis, the release of vasoactive substances (LPS and histamine) leads to vasoconstriction and dilation, resulting in the destruction of the microvasculature of the corium [19,20]. The corium—rich in vascularity and neurons—nourishes the dermal lamellae between the lamina and the distal phalanx. Evidence shows that the levels of LPS, histamine, lactic acid, and other substances in the body mainly come from the metabolism of gut microbiota [21,22,23]. In addition, previous studies have indicated that changes in rumen microflora and their metabolites induced by high-grain or high-sugar diets play an important role in the development of laminitis [14,24]. However, the alterations in the rumen bacterial community and its main metabolites are still not clearly understood in clinical and naturally occurring cases of laminitis in dairy cows. Therefore, we collected the rumen fluid samples of cows with laminitis to analyze its characteristics and provide a reference for the development of microecological preparations to treat laminitis.

## 2. Materials and Methods

### 2.1. Farms and Animals

A cross-sectional study was carried out at smallholder dairy farms located in Linqu Country, Weifang City, Shandong Province, China. The cows were obtained from the same farm. The enclosure and bed in the farm were relatively clean, and the feces and urine were cleaned in a timely manner. In addition, the TMR was composed of forage and concentrates showed in Table 1. The cows were fed daily at 5:00 and 18:00, and had free access to water. Animals were selected by lameness examination and divided into control and lameness groups. Lameness bovines were further examined by shoeing to identify laminitis. The diagnosis of laminitis, as previously described, and the scoring criteria are shown in Table 2 [25]. We chose the scores 2–4 in the present study. In all, eight Holstein cows were identified as having laminitis, and eight healthy cows were used as controls (average body weight = 552 ± 73 kg, at mid-lactation, the feed components were the same and the weights were similar). After feeding for 4 to 5 h, blood samples were collected from the jugular vein and centrifuged at 3000× *g* at 4 °C for 30 min. Plasma samples were divided into two portions. One portion was collected and transferred into a sterile, depyrogenated glass tube, and kept at −20 °C for LPS detection. The other portion was transferred to a 1.5 mL centrifuge tube and place at −20 °C for lactic acid and histamine detection. In addition, the rumen fluid samples were harvested through an inverted tube. The samples were filtered through 2 layers of gauze and the pH was immediately measured with a portable pH meter, and then frozen in liquid nitrogen and kept at −80 °C for 16S rDNA gene amplicon pyrosequencing.

### 2.2. Lipopolysaccharide Concentration Detection

The plasma was quantified using a chromogenic endpoint assay (Chinese Horseshoe Crab Reagent Manufactory Co., Ltd., Xiamen, China) with a minimum detection limit of 0.01 EU/mL under the manufacturer’s instructions.

### 2.3. Lactic Acid and Histamine Concentrations Detection

The plasma detected the concentrations of lactic acid and histamine using the detection kits, according to the manufacturer’s instructions (Jiangsu feiya Biological Technology, Suzhou, China).

### 2.4. DNA Extraction, Illumina MiSeq Sequencing, Bioinformatics Analyses

The genome DNA of ruminal fluid was extracted using a CTAB/SDS method. The DNA concentration and purity were detected by 1% agarose gels. To amplify the 16S rDNA, barcoded primers (16S V4:515F-806R) targeting the V4 region were used. The PCR reactions were conducted with Phusion^®^ High-Fidelity PCR Master Mix (New England Biolabs, Ipswich, MA, USA). PCR products were mixed in equal ratios and then purified with a Qiagen Gel Extraction Kit (Qiagen, Germany). Sequencing libraries were generated using the TruSeq^®^ DNA PCR-Free Sample Preparation Kit (Illumina, San Diego, CA, USA). The library quality was evaluated by a Qubit@ 2.0 Fluorometer (Thermo Scientific, Waltham, MA, USA) and an Agilent Bioanalyzer 2100 system. Finally, the library was sequenced on an Illumina HiSeq 2500 platform, and 250 bp paired-end reads were generated. Single-end reads were performed according to the unique barcode and truncated by cutting off the barcode and primer sequence of the samples. The high-quality clean reads were acquired according to the cutadapt quality controlled process based on the quality filtering of the raw reads that were conducted in the specific filtering conditions. Furthermore, sequences with similarity ≥ 97% were assigned to the same OTUs, and the representative sequence of each OTU was screened for further annotation.

In addition, bacterial community diversity and richness were analyzed by ace, chao 1, the Shannon index, the Simpson index, and the observed species. The distance of bacterial community between control and laminitis groups was evaluated by the NMDS of Bray–Curtis dissimilarity. The bacterial taxa between control and laminitis groups were evaluated by LEfse, and a Venn diagram was used to evaluate the numbers of core genera in the ruminal contents from the control and the laminitis groups.

### 2.5. Statistical Analysis

Statistical analysis was conducted by GraphPad Prism 6.01 (GraphPad Software, Inc., San Diego, CA, USA). All data are presented as Means ± SEM. To compare differences between various experimental groups, a two-tailed *t*-test were used. A *p* < 0.05 or *p* < 0.01 was considered statistical significance.

## 3. Results

### 3.1. LPS, Lactic Acid, and Histamine in Plasma

**Figure 1:**
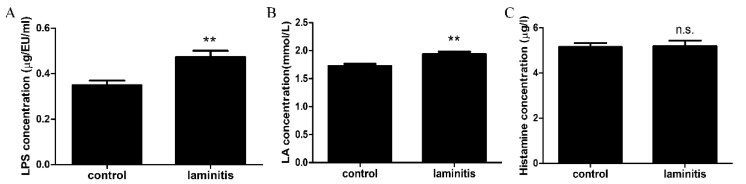
LPS, lactic acid, and histamine in plasma the concentration of (**A**) LPS, (**B**) lactic acid, and (**C**) histamine in plasma between control and laminitis cows. ** *p* < 0.01 are significantly different from laminitis group. n.s. means there is no significant difference between the two groups.

As shown in Figure 1A–C, the concentration of LPS and lactic acid in plasma from laminitis group cows was significantly increased compared to the control group cows, while there was no difference in histamine in the plasma.

### 3.2. PH in Rumen Fluid

As shown in Table 3, the pH in rumen fluid of laminitis cows was 5.82, which was lower than that of healthy cows (rumen fluid pH = 6.2).

### 3.3. The Composition of the Ruminal Bacterial Community

Next, 16S-rDNA sequencing was utilized to observe alterations in the microbiota in the control and laminitis cows. In total, 1,196,161 gene sequences were detected from ruminal fluid samples, with an average of 74,760 sequences per sample. Rarefaction curves showed that most of the bacterial diversity had sufficient sequences, as proven by the sampling depth (Supplementary Figure S1).

**Figure 2:**
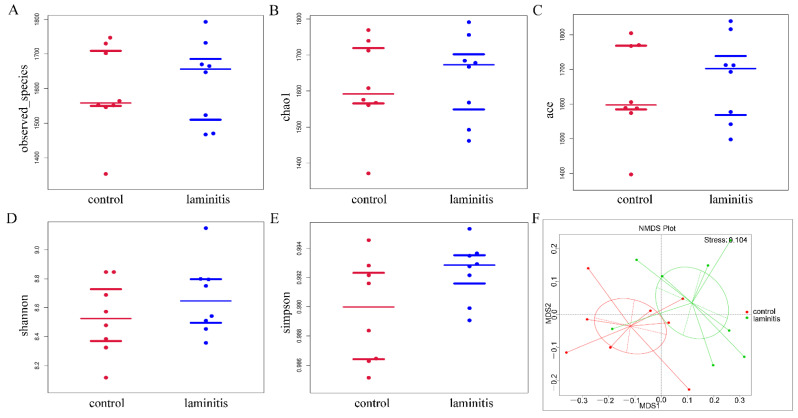
The composition of ruminal bacterial community comparison of the rumen microbiota richness and diversity in terms of (**A**) observed species, (**B**) chao 1, (**C**) ace, (**D**) Shannon index, and (**E**) Simpson index; (**F**) non-metric multidimensional scaling (NMDS) plot of pair wise Bray–Curtis dissimilarities between control and laminitis cows.

When comparing the control group with the laminitis group, there were no significant differences in community richness and diversity, as shown by observed species, chao 1, and ace, as well as the Shannon index and the Simpson index (Figure 2A–E). In addition, the non-metric multidimensional scaling (NMDS) ordination showed that there was a separation of the bacterial community between the control and laminitis groups using Bray–Curtis dissimilarity (Figure 2F).

### 3.4. Changes in Ruminal Bacterial Community at the Phylum Level

**Figure 3:**
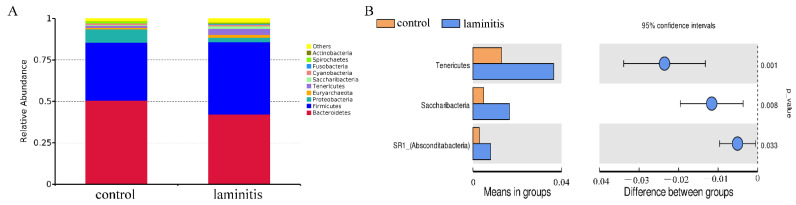
Changes in ruminal bacterial community at the phylum level (**A**) relative abundances of the top 10 phyla of rumen fluid between control and laminitis group cows. *Bacteroidetes*, *Firmicutes*, and *Proteobacteria* were the most abundant phyla in ruminal bacterial community; (**B**) a *t*-test showed that the relative abundances of *Tenericutes*, *Saccharibacteria*, and SR1 were significantly increased in laminitis cows compared to the control group.

At the phylum level, the bacterial sequences obtained from all cows were comprised of 21 phyla, which was the same in both groups. Among them, *Bacteroidetes* (control vs. laminitis, 50.64% vs. 42.28%) and *Firmicutes* (34.85% vs. 43.64%) were the most abundant phyla in the ruminal bacterial community. These were followed by *Proteobacteria* (8.03% vs. 2.66%), *Spirochaetes* (1.08% vs. 0.48%), *Tenericutes* (1.29% vs. 3.65%), and *Euryarchaeota* (0.86% vs. 1.66%) (Figure 3A). The results of the *t*-test showed that the relative abundances of *Tenericutes*, *Saccharibacteria*, and SR1 (*Absconditabacteria*) were significantly increased in laminitis cows compared to the control group (Figure 3B).

### 3.5. Changes in Ruminal Bacterial Community at the Genus and Species Levels

**Figure 4:**
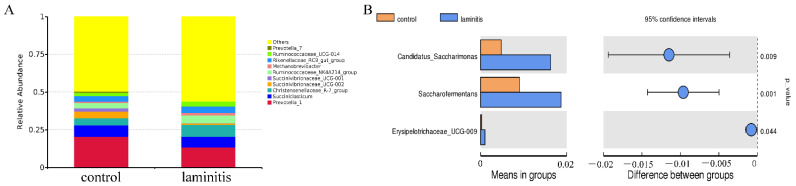
Changes in ruminal bacterial community at the genus level (**A**) relative abundances of the top 10 genera of rumen fluid between control and laminitis group cows. *Prevotella 1*, *Succiniclasticum*, *Succinivibrionaceae UCG-002*, *Christensenellaceae R-7-group*, *Succinivibrionaceae UCG-001*, *Ruminococcaceae NK4A214-group*, *Ruminococcaceae UCG-014*, and *Rikenellaceae mRC9* gut group were the most abundant genera in the ruminal bacterial community; (**B**) a *t*-test showed that the relative abundances of *Candidatus Saccharimonas*, *Saccharofermentans*, and *Erysipelotrichaceae UCG-009* were significantly increased in the laminitis group.

At the genus level, the bacterial sequences detected from all animals were comprised of 245 genera. The results obtained from preliminary analysis of the dominant genera are shown in Figure 4A as *Prevotella 1* (control vs. laminitis, 20.44% vs. 13.46%), *Succiniclasticum* (7.77% vs. 7.06%), *Succinivibrionaceae UCG-002* (4.34% vs. 0.79%), *Christensenellaceae R-7-group* (4.63% vs. 7.94%), *Succinivibrionaceae UCG-001* (2.20% vs. 0.30%), *Ruminococcaceae NK4A214-group* (3.52% vs. 5.11%), *Ruminococcaceae UCG-014* (2.12% vs. 3.29%), and *Rikenellaceae mRC9-*gut group (3.85% vs. 4.24%). The *t*-test showed that the relative abundances of *Candidatus Saccharimonas*, *Saccharofermentans*, and *Erysipelotrichaceae UCG-009* were significantly increased in the laminitis group; No differences in the other genera were statistically significant (Figure 4B).

**Figure 5:**
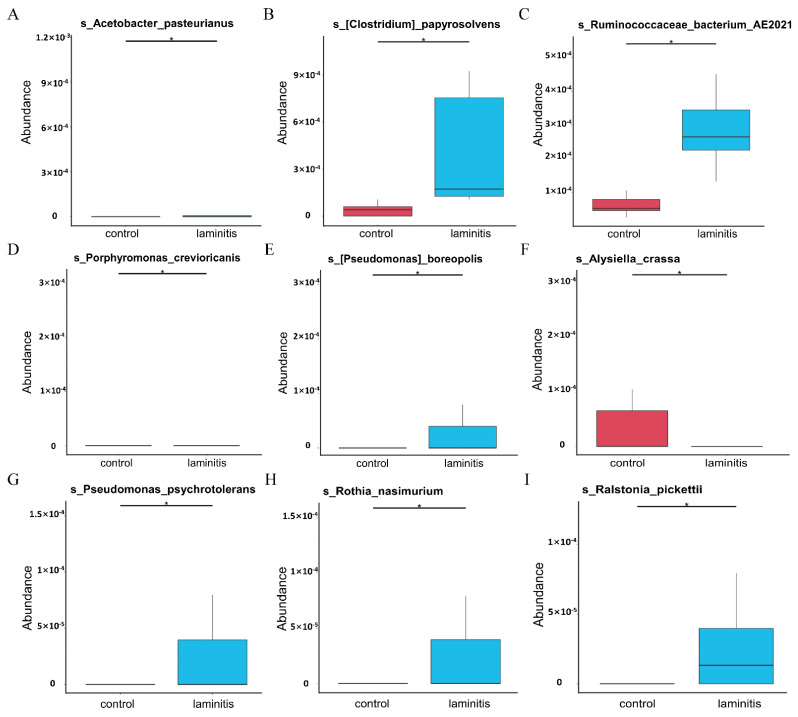
MetaStat analysis The relative abundance of (**A**) *Acetobacter pasteurianus*, (**B**) *Clostridium papyrosolves*, (**C**) *Ruminococcaceae bacterium AE2021*, (**D**) *Porphyromonas crevioricanis*, (**E**) *Pseudomonas boreopolis*, (**F**) *Alysiella_crassa*, (**G**) *Pseudomonas psychrotolerans*, (**H**) *Rothia nasimurium*, and (**I**) *Ralstonia pickettii*. * *p* < 0.05 is significantly different from laminitis group.

**Figure 6:**
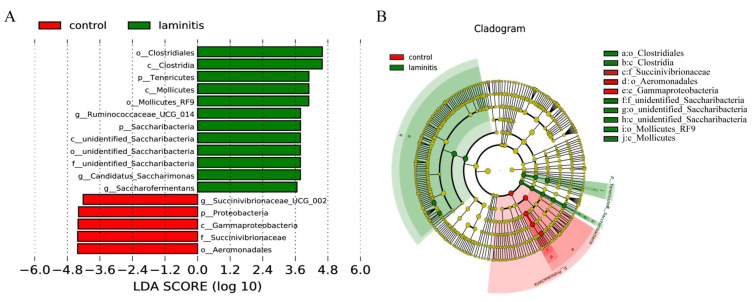
Taxonomic biomarkers: (**A**) LEfSe analysis between the control (red) and laminitis (green) groups. *Ruminococcaceae UCG 014*, *Candidatus Saccharimonas*, and *Saccharofermentans* genera were enriched in laminitis cows, while *Succinivibrionaceae_UCG-002* genus was enriched in control group cows. (**B**) Cardiogram showing differentially abundant taxonomic clades with an LDA score  >  3.5 among laminitis and control groups, *p*  <  0.05.

At the species level, the MetaStat analysis showed that the species of *Acetobacter pasteurianus*, *Clostridium papyrosolvens*, *Ruminococcaceae bacterium AE2021*, *Porphyromonas crevioricanis*, *Pseudomonas boreopolis*, *Pseudomonas psychrotolerans*, *Rothia nasimurium*, and *Ralstonia pickettii* increased significantly, while the relative abundance of *Alysiella_crassa* decreased significantly in the ruminal microbiota from the laminitis samples compared to the control cows (Figure 5A–I). Furthermore, a biomarker analysis by linear discriminant analysis (LDA) effect size (LEfSe) and a cladogram generated from the LEfSe analysis on the microbiota community of rumen showed that the *Ruminococcaceae UCG 014*, *Candidatus Saccharimonas*, and *Saccharofermentans* genera were enriched in the laminitis cows (Figure 6A,B).

## 4. Discussion

Bovine laminitis, one of the most costly lameness conditions, is an economic drain on producers [26]. It is generally accepted that the micro-circulation of a blood disorder within the corium induced by microbiota metabolites, such as LPS, lactate, and histamine, is the main pathogenesis of laminitis [10,11,12]. Thus, in the present study, we detected the characteristics of rumen microbiota and the concentration of LPS, lactic acid, and histamine in the plasma from cows from control and laminitis groups. The results showed that the concentrations of LPS and lactic acid in plasma were significantly increased in the laminitis cows compared to the control cows (0.47 vs. 0.35 µg/EU/mL, 1.93 vs. 1.72 mmol/L). In addition, the elevated abundance of bacteria, including *Candidatus Saccharimonas*, *Saccharofermentans*, *Erysipelotrichaceae UCG-009*, *Acetobacter pasteurianus*, *Clostridium papyrosolvens*, and *Porphyromonas crevioricanis*, which enrich acid-enhancing metabolites could lower the pH of rumen fluid, leading to the death of Gram-negative bacteria and the release of endotoxins in rumen. However, to some extent, the small sample size limits the generalization of the findings of this study.

LPS is a major component of the outer membrane of Gram-negative bacteria. As the main vasoactive substance, LPS plays a key role in inflammation [27]. When ruminal acidosis occurs, plenty of Gram-negative bacteria are dead and the LPS is released. In the early stage of laminitis, inflammation is the main manifestation of the disease. LPS is absorbed into blood circulation through the ruminal wall, and it then reaches the micro-circulation of the claw. Local LPS has inflammatory effects, such as the activation of cytokines and acute-phase protein release, thrombocytopenia, leukopenia, followed by leukocytosis [20]. Moreover, recent evidence suggests that pathological changes in inflammation could be initiated by the injection of LPS [18]. In this study, the concentration of LPS was significantly increased in the laminitis group compared with the control group. In addition, lactic acid, as vasoactive a substance as LPS, is associated with laminitis [28]. Previous studies have reported the increase in histamine and lactic acid in bovine serum during laminitis [3]. Our results are similar to those in previous studies and have confirmed that lactic acid the in plasma of bovines with laminitis increases.

The ruminal bacterial community plays a crucial role in pathologic regulation of organisms when developing diseases [29]. The role of microbial populations has received widespread attention across several disciplines in recent years. Therefore, accumulating studies suggest there is an association among microbiota, its metabolites, and laminitis [14]. The proportion of phylum *Firmicutes* and genera *Streptococcus* and *Lactobacillus* was significantly increased, while the abundances of phyla *Bacteroidetes* and *Fibrobacteres* and genera *Butyrivibrio* and *Ruminococcus* dramatically reduced during laminitis [28]. Our results show that the relative abundances of *Tenericutes*, *Saccharibacteria*, and SRI phyla were markedly increased from rumen fluid in laminitis cows compared to control cows. This may be due to the different diets of the animals. Our study also collected rumen fluid samples from healthy and laminitis cows being fed the same diet, whereas other studies utilized high carbohydrate feeding to induce animal models of laminitis [14,24].

In the present study, we observed significant differences in the bacterial community between the control and laminitis groups using NMDS. In addition, compared to the control group, the relative abundances of *Candidatus Saccharimonas*, *Saccharofermentans*, and *Erysipelotrichaceae UCG-009* genus increased. Evidence shows that *Candidatus_Saccharimonas* was positively correlated with ruminal propionate concentrations in dairy cows [30]. *Saccharofermentans* belongs to the *Bacteroidetes* phylum and participates in hemicellulose, pectin, arabinogalactan, starch, fructan, and chitin degradation [31]. In addition, this study showed that *Erysipelotrichaceae UCG-009* was positively associated with the production of butyrate [32]. At the species level, *Acetobacter pasteurianus*, *Clostridium papyrosolvens*, *Ruminococcaceae bacterium AE2021*, *Porphyromonas crevioricanis*, *Pseudomonas boreopolis*, *Pseudomonas psychrotolerans*, *Rothia nasimurium*, and *Rothia pickettii* increased, while the relative abundance *Alysiella_crassa* was reduced. *Acetobacter pasteurianus*, a member of *Alphaproteobacteria*, is an acetic acid-producing bacterium. *Acetobacter pasteurianus* is usually present in sugar-rich substrates such as fruits, flowers, and vegetables [33]. *Clostridium papyrosolvens* can produce a wide variety of carbohydrate-active enzymes to enhance cellulosic biomass degradation [34]. *Ruminococcaceae* have been suggested to participate in bile acid metabolism [35]. *Porphyromonas crevioricanis*, an anaerobic, non-spore-forming, and Gram-negative bacillus, can produce butyric and phenylacetic acids [36]. *Rothia nasimurium* are commonly found as commensal bacteria in the upper respiratory tract and gut of humans and other animals, and have received attention for their multidrug and pathogenic applications [37,38]. These results suggest that the elevated abundance of bacteria, which enriches acid-enhancing metabolites, may decrease pH values of the rumen liquid, destroying the rumen microenvironment. As such, harmful metabolites, such as LPS and lactic acid, could be produced in large quantities and enter the circulatory system to reach the hoof and cause damage. Thus, changing flora may be associated with the development of laminitis.

## 5. Conclusions

This study shows that vasoactive substances, such as LPS and lactic acid, are associated with laminitis. In addition, there were differences in the ruminal bacterial community between control and laminitis cows. The increased abundances of *Candidatus Saccharimonas*, *Saccharofermentans*, and *Erysipelotrichaceae UCG-009* genera, as well as *Acetobacter pasteurianus*, *Clostridium papyrosolvens*, *Ruminococcaceae bacterium AE2021*, *Porphyromonas crevioricanis*, *Pseudomonas boreopolis*, *Pseudomonas psychrotolerans*, *Rothia nasimurium*, and *Rothia pickettii* species in rumen may be associated with the development of laminitis in dairy cows. Given these results, targeting ruminal microbiota may be a vital approach to prevent laminitis in cows.

## Figures and Tables

**Table 1:** Dietary composition of dairy cows.

Item	Percentage (%) of Ingredients
Beet pulp	3.59
Cottonseed	1.54
Alfalfa	10.25
Ensiling	51.24
Bean pulp	11.27
Extruded soybean	1.28
Maize	17.68
Fatty powder	0.92
1% gunk	0.26
Mineral additive	1.97

**Table 2:** Lameness score in cattle [25].

Score	Name	Description
1	Normal	Straight back when standing in quadrupedal position and walking. Normal step.
2	Mild lameness	Straight back quadrupedal and arched when walking. Normal step.
3	Moderate lameness	Arched ack when standing and walking. Shortened step of one or more members.
4	Evident lameness	Arched back when standing and walking. Locomotion changed with one step at a time or avoiding the support of a limb.
5	Severe lameness	In addition to previous signs, the calves are reluctant or have difficulty supporting one or more limbs even when standing.

**Table 3:** PH in rumen fluid.

	Control	Laminitis
PH in rumen fluid	6.2 ± 0.17	5.82 ± 0.7 *

* *p* < 0.05 is significantly different from laminitis group.

## Data Availability

The data presented in this study are available on request from the corresponding author.

## Supplementary Materials

The following are available online at https://www.mdpi.com/article/10.3390/genes12121996/s1, Supplementary Figure S1. Rarefaction curves comparing the number of reads with the number of phylotypes found in the DNA from rumen fluid of healthy bovines and laminitis bovines.
