# Supplementary material for: Characterization of the Bacterial Community of Rumen in Dairy Cows with Laminitis
Source: Genes (Basel). 2021 Dec 16;12(12):1996. doi: 10.3390/genes12121996 (PMC8700892; doi:10.3390/genes12121996)
Supplement: Supplementary file 1 [file genes-12-01996-s001.zip › genes-1467656-supplementary.pdf]

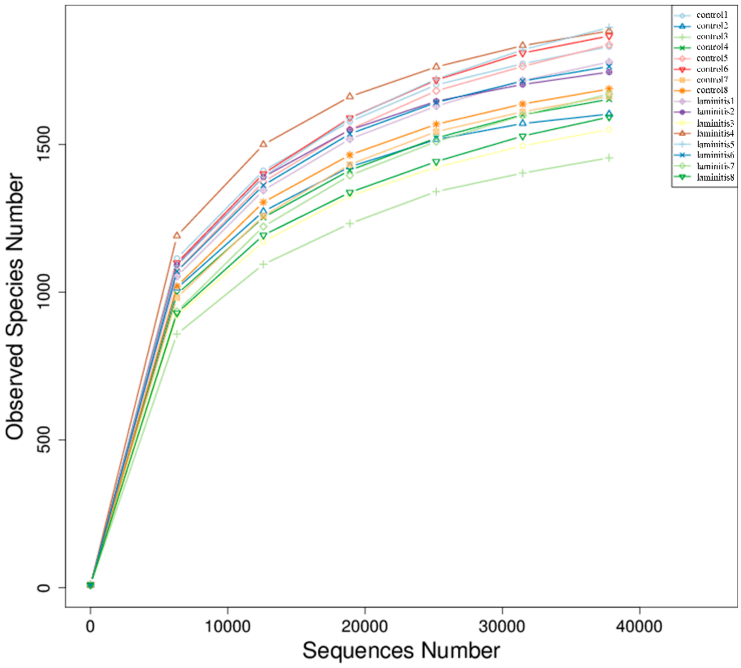

Figure S1. Rarefaction curves comparing the number of reads with the number of phylotypes found in the DNA from rumen fluid of healthy bovines and laminitis bovines.
